# Corrections

**DOI:** 10.1016/S2215-0366(17)30514-X

**Published:** 2018-02

*Fazel S, Ramesh T, Hawton K. Suicide in prisons: an international study of prevalence and contributory factors. Lancet Psychiatry* 2017; **4:** 946–52—In figure 1 of this Article, the p values were not listed in the correct order. This correction has been made to the online version as of Dec 21, 2017.

